# Improving diagnostic performance of the Kato-Katz method for *Clonorchis sinensis* infection through multiple samples

**DOI:** 10.1186/s13071-019-3594-5

**Published:** 2019-07-08

**Authors:** Men-Bao Qian, Shi-Feng Zhuang, Shi-Qiao Zhu, Xiao-Mao Deng, Zheng-Xiang Li, Xiao-Nong Zhou

**Affiliations:** 1 National Institute of Parasitic Diseases, Chinese Center for Disease Control and Prevention, Shanghai, 200025 China; 2 Chinese Center for Tropical Diseases Research, Shanghai, 200025 China; 3 Key Laboratory of Parasite and Vector Biology, Ministry of Health, Shanghai, 200025 China; 4 WHO Collaborating Center for Tropical Diseases, Shanghai, 200025 China; 5 Hunan Center for Disease Control and Prevention, Changsha, 410005 China; 6 Qiyang Center for Disease Control and Prevention, Qiyang, 426100 China

**Keywords:** *Clonorchis sinensis*, Clonorchiasis, Kato-Katz method, Diagnosis

## Abstract

**Background:**

Clonorchiasis is caused by eating of raw or undercooked freshwater fish containing the larvae of *Clonorchis sinensis*; the Kato-Katz method is widely applied in diagnosis. The improvement of repeated Kato-Katz smears from multiple stool samples has been well illuminated in many helminths other than *C. sinensis*.

**Methods:**

A cross-sectional investigation was implemented to capture the epidemiology and risk factors of clonorchiasis among middle school students in Qiyang county, China. Students with complete data of six Kato-Katz thick smears from two stool samples were included in this analysis. Data on the habits of eating raw freshwater fish were also collected and compared.

**Results:**

Altogether, 397 students had complete information of six smears, out of which 394 reported the information on eating habits. According to the ‘gold’ standard by six smears, 77 students (19.4%) were detected with *C. sinensis*. However, only 45 (11.3%) were detected using a single smear, with an underestimation of 41.6% compared to the ‘gold’ standard. However, the geometric mean of eggs per gram of feces in detected cases was 126.4 in a single smear, overestimated by 105.2% compared to 61.6 by the ‘gold’ standard. The linear relationship between prevalence and infection intensity of detected cases based on different smears was significantly negative. The habits of eating raw freshwater fish in the false negative cases was similar to those in the detected cases, but these two groups had significantly higher levels for habits of eating raw freshwater fish than negative individuals.

**Conclusions:**

In low endemicity situations, underestimation of *C. sinensis* infection could not be avoided based on a limited number of Kato-Katz smears. Thus, repeated smears from at least two stool samples should be considered when an individual eats raw freshwater fish, drug efficacy is evaluated or elimination of *C. sinensis* is verified. Additionally, when logistics are insufficient for multiple samples to be taken for diagnosis for survey and surveillance in the areas or populations of low endemicity, prevalence accuracy needs to be corrected.

## Background

Clonorchiasis is caused by eating raw or undercooked freshwater fish, which contains infective larvae of *Clonorchis sinensis* [1]. Clonorchiasis is highly endemic in Asia, including China, the Republic of Korea, Vietnam and part of Russia, due to their popular habits of eating raw freshwater fish [2–6]. There are about 15 million people implicated with *C. sinensis* infection, out of which about 13 million are domiciled in China [2, 3]. *Clonorchis sinensis* infection exerts a significant disease burden due to the high comorbidity of gallstone, cholangitis, cholecystitis and especially cholangiocarcinoma [7–9].

Accurate diagnosis for clonorchiasis is important because it determines the drug treatment for the infected individual [1, 10]. On the other hand, accurate diagnosis of individuals could impact the assessment of endemicity, which will then determine the choices of intervention measures and subsequent evaluation of effectiveness [10]. Similar to other helminthiases, the detection of eggs in a stool is a definite diagnosis [1]. The Kato-Katz method, formalin-ether concentration technique and direct smear method are usually used for diagnosis among the fecal examination techniques [11, 12]. Owing to the simplicity of use and capacity for quantification, the Kato-Katz method is frequently applied in large epidemiological surveys, the evaluation of drug efficacy, monitoring and evaluation of intervention, etc. [3, 13, 14]. Due to the variation in the way helminths lay eggs and the deficiency of diagnostic techniques, false negative cases usually cannot be completely avoided in stool examination, especially when the number of observed eggs in the stool is low [15–19]. Thus, the strategy of increasing the number of smears from multiple stool samples is usually applied to increase sensitivity, which has been effectively demonstrated in many helminths including soil-transmitted helminths, *Schistosoma* spp. and *Opisthorchis viverrini* [15–19]. In a previous study, we demonstrated that the Kato-Katz method accuracy is highly improved by increasing the number of smears and stool samples of the individuals with *C. sinensis* infection after drug treatment [20]. However, the performance of this strategy has not been assessed in a natural setting with *C. sinensis* infection.

In the present study, we evaluated the diagnostic performance of the Kato-Katz method in *C. sinensis* infection by increasing the number of smears and stool samples in a natural setting. Additionally, we included information on eating of raw freshwater fish into the study to explore its relationship with diagnostic performance. Meanwhile, the diagnostic performance in soil-transmitted helminth infections was also analyzed.

## Methods

### Study design and study area

This study was embedded in a cross-sectional study to explore the epidemiology and risk factors of clonorchiasis in middle school children in Qiyang county, Hunan province in 2013. Hunan province is located in southeastern China and Qiyang county is located in the south of Hunan province. A highly endemic town named Dazhongqiao was selected based on a previous survey [21]. The town had a total population of about 50,000 in 2013. The town had two middle schools, with 473 and 172 students, respectively. All students from the two middle schools were eligible.

### Investigation procedures

Each student was asked to provide two stool samples. Furthermore, a questionnaire was administered to each participant to collect information, including whether or not they ate raw freshwater fish. The stool samples were transferred to the local Center for Disease Control and Prevention. The Kato-Katz method using 41.7 mg templates was employed to examine for the presence of helminth eggs in the stool and three thick smears for each sample were prepared and then read [22]. The helminth eggs obtained were recorded both qualitatively and quantitatively.

### Statistical analysis

Only students with complete data of six smears from two stool samples were included in this analysis. The diagnostic ‘gold’ standard was defined as a combination of six Kato-Katz thick smears from two stool samples. The prevalence was defined as the proportion of the number of students with infection to the total number of students enrolled and 95% confidence intervals (95% CI) were calculated. The false negative rate was defined as the proportion of the number of cases wrongly classified as negative by a ‘non-gold’ standard in the total number of cases diagnosed by the ‘gold’ standard. Two different algorithms were applied to calculate the infection intensity in terms of eggs per gram of feces (EPG) per individual. First, the arithmetic average of the number of eggs in available smears by the ‘non-gold’ standard was multiplied by 24. Secondly, the arithmetic average of the number of eggs in all six smears by the ‘gold’ standard was multiplied by 24. Correspondingly, the geometric mean of EPG (GMEPG) of the positive cases was provided through two algorithms. However, only the second algorithm could be applied to calculate the GMEPG in the false negative cases. Student’s t-test was applied to compare the EPG after logarithmic transformation in the positive and the false negative cases. Linear regression analysis was used to test the relationship between the prevalence and infection intensity of the positive or the false negative cases based on different diagnostic standards and the coefficient of determination (*R*^2^) was provided.

In *C. sinensis* infection, the proportion of participants eating raw freshwater fish was calculated for different groups and Pearson’s Chi-square test was applied to test the difference. When Pearson’s Chi-square test could not be used, Fisher’s exact test was used instead.

## Results

### Overall information

Altogether, 557 students (10–17 years-old) provided at least one stool sample. Among them, 397 students had complete data of six thick smears from two stool samples, of which 394 provided information on eating practices. Among these 397 students, 77, 20, 3 and 0 had *C. sinensis*, *Trichuris trichiura*, *Ascaris lumbricoides* and hookworm infections, respectively. Thus, the diagnostic performance in *C. sinensis* and *T. trichiura* was analyzed.

### Diagnostic accuracy of clonorchiasis

Based on the ‘gold’ standard, 77 out of 397 students were detected as infected with *C. sinensis* (prevalence of 19.4%; 95% CI: 15.5–23.3%) (Table 1). The diagnostic performance improved following an increase in the number of smears. The prevalence was only 11.3% (95% CI: 8.2–14.5%) in a single smear from the first sample, which was significantly lower than that of the ‘gold’ standard. It was also significantly lower than that of four smears from two samples, which gave a prevalence of 18.4% (95% CI: 14.6–22.2%). Compared to the ‘gold’ standard, the false negative rate was 41.6% in a single smear. The false negative rate decreased following an increase in the number of smears.

**Table 1:** Diagnostic performance of the Kato-Katz method for *C. sinensis* infection

No. of smears	No. of positive cases	No. of negative cases	Prevalence (95% CI) (%)	False negative rate (%)	GMEPG of the positive cases (1)^a^	Overestimation of GMEPG (%)^b^	GMEPG of the positive cases (2)^c^	GMEPG of the false negative cases^c^
1 stool × 1 smear	45	352	11.3 (8.2–14.5)	41.6	126.4	105.2	121.2	23.8^d^
1 stool × 2 smears	49	348	12.3 (9.1–15.6)	36.4	119.9	94.7	100.8	26.0^e^
1 stool × 3 smears	55	342	13.9 (10.4–17.3)	28.6	91.0	47.8	79.0	33.1^f^
2 stools × 1 smear	64	333	16.1 (12.5–19.8)	16.9	95.4	55.0	96.1	6.9^g^
2 stools × 2 smears	73	324	18.4 (14.6–22.2)	5.2	74.1	20.3	70.2	5.7^h^
2 stools × 3 smears	77	320	19.4 (15.5–23.3)	0.0	61.6	0.0	61.6	–

^a^Only the available smears were calculated

^b^The calculation was based on the GMEPG of the positive cases (1)

^c^All six smears in ‘gold’ standard were calculated

^d^*t*_(75)_ = 4.39, *P* < 0.001, compared to the GMEPG of the positive cases (2)

^e^*t*_(75)_ = 3.42, *P* = 0.001, compared to the GMEPG of the positive cases (2)

^f^*t*_(75)_ = 1.97, *P* = 0.053, compared to the GMEPG of the positive cases (2)

^g^*t*_(75)_ = 9.62, *P* < 0.001, compared to the GMEPG of the positive cases (2)

^h^*t*_(75)_ = 2.87, *P* = 0.005, compared to the GMEPG of the positive cases (2)

Following an increase in the number of smears, the GMEPG of the positive cases decreased from 126.4 to 61.6 based on available smears, which showed an overestimation by 105.2% in single smear compared to that in the ‘gold’ standard (Table 1). Correspondingly, the GMEPG of the positive cases calculated by six smears decreased from 121.2 to 61.6. Overall, the GMEPG of the false negative cases showed a declining trend. The intensity in the false negative cases was lower than that in the positive cases.

**Fig. 1:**
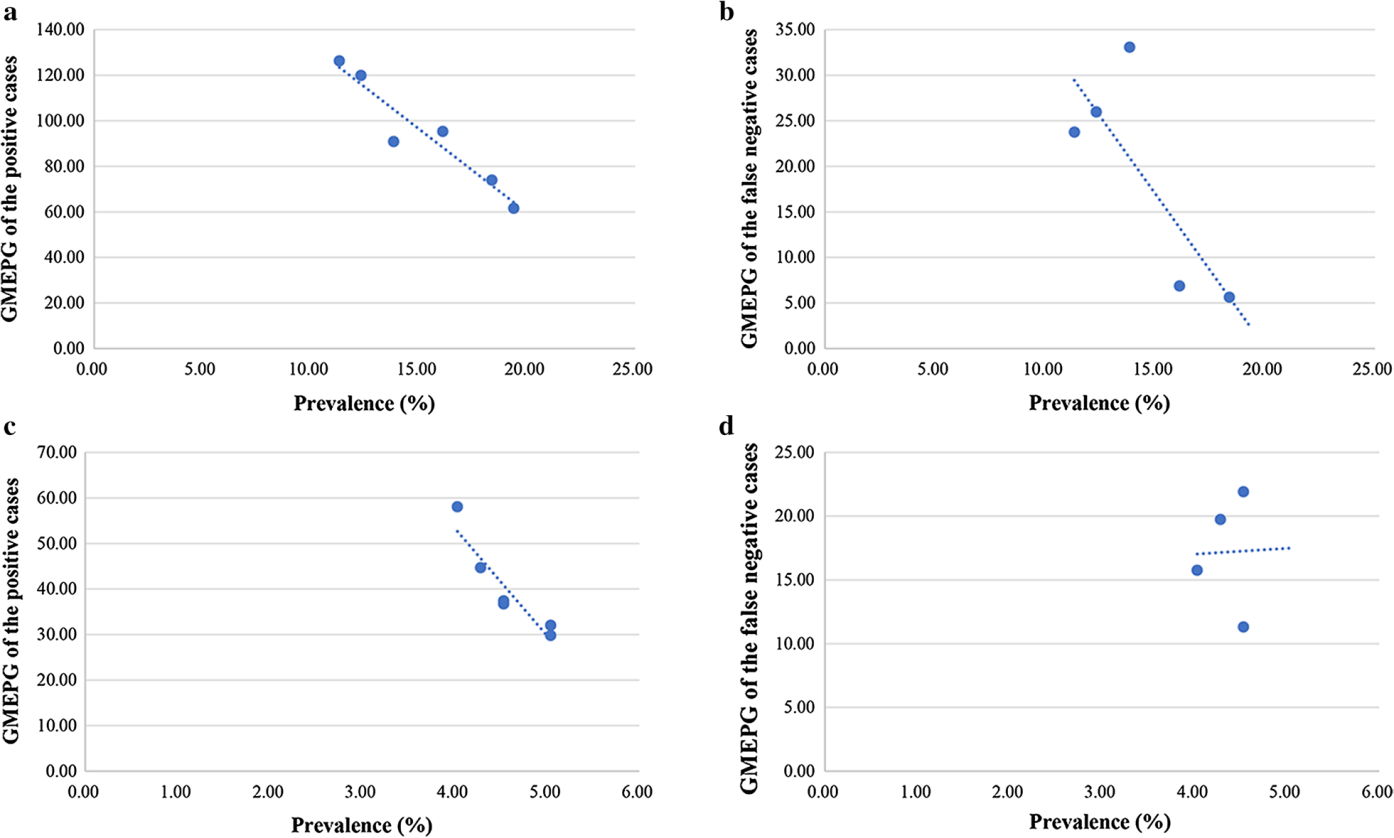
The relationship between prevalence and infection intensity based on different diagnostic standards. **a** The prevalence and infection intensity of the positive cases based on available smears in *C. sinensis* infection. **b** The prevalence and infection intensity of the false negative cases based on six smears in *C. sinensis* infection. **c** The prevalence and infection intensity of the positive cases based on available smears in *T. trichiura* infection. **d** The prevalence and infection intensity of the false negative cases based on six smears in *T. trichiura* infection

The linear relationship between the prevalence and the intensity of the positive cases based on available smears was significantly negative (*R*^2^ = 0.911, *P* = 0.003) (Fig. 1a). We observed a negative relationship trend between the prevalence and intensity of the false negative cases based on six smears, but it was not significant (*R*^2^ = 0.630, *P* = 0.109) (Fig. 1b).

### Eating raw freshwater fish and the diagnostic performance

The proportion of *C. sinensis*-infected students as confirmed with the ‘gold’ standard who ate raw freshwater fish was 70.7%. This proportion in the positive cases based on different diagnostic standards did not change significantly, varying between 68.3–73.6% (Table 2). The proportion varied between 25.1–28.9% in the negative cases based on different diagnostic standards, which were all significantly lower than that of the positive cases. The proportion in the false negative cases based on different diagnostic standards who ate raw freshwater fish varied between 63.6–100.0%, which were also all higher than the proportion in the negative cases but not significantly different from the proportion in the positive cases.

**Table 2:** The proportion eating raw freshwater fish in different groups of *C. sinensis* infection based on different diagnostic standards

No. of smears	Positive cases				Negative cases				False negative cases			
	*N*	Consuming raw fish	Not consuming raw fish	Unclear data	*N*	Consuming raw fish	Not consuming raw fish	Unclear data	*N*	Consuming raw fish	Not consuming raw fish	Unclear data
		*n* (%)^a^	*n*	*n*		*n* (%)^a^	*n*	*n*		*n* (%)^a^	*n*	*n*
1 stool × 1 smear	45	32 (72.7)^b^	12	1	352	101 (28.9)^c^	249	2	32	21 (67.7)^d^	10	1
1 stool × 2 smears	49	35 (72.9)^e^	13	1	348	98 (28.3)^f^	248	2	28	18 (66.7)^g^	9	1
1 stool × 3 smears	55	39 (73.6)^h^	14	2	342	94 (27.6)^i^	247	1	22	14 (63.6)^j^	8	0
2 stools × 1 smear	64	43 (68.3)^k^	20	1	333	90 (27.2)^l^	241	2	13	10 (83.3)^m^	2	1
2 stools × 2 smears	73	50 (69.4)^n^	22	1	324	83 (25.8)^o^	239	2	4	3 (100.0)^p^	0	1
2 stools × 3 smears	77	53 (70.7)^q^	22	2	320	80 (25.1)	239	1	0	0	0	0

^a^No. with unclear data was not included in the calculation of the proportion

^b^*χ*^2^ = 33.64, *df* = 1, *P* < 0.001, positive cases *vs* negative cases

^c^*χ*^2^ = 19.78, *df* = 1, *P* < 0.001, negative cases *vs* false negative cases

^d^*χ*^2^ = 0.22, *df* = 1, *P* = 0.641, positive cases *vs* false negative cases

^e^*χ*^2^ = 37.49, *df* = 1, *P* < 0.001, positive cases *vs* negative cases

^f^*χ*^2^ = 17.18, *df* = 1, *P* < 0.001, negative cases *vs* false negative cases

^g^*χ*^*2*^ = 0.33, *df* = 1, *P* = 0.568, positive cases *vs* false negative cases

^h^*χ*^2^ = 43.44, *df* = 1, *P* < 0.001, positive cases *vs* negative cases

^i^*χ*^*2*^ = 12.87, *df* = 1, *P* < 0.001, negative cases *vs* false negative cases

^j^*χ*^2^ = 0.74, *df* = 1, *P* = 0.389, positive cases *vs* false negative cases

^k^*χ*^2^ = 39.91, *df* = 1, *P* < 0.001, positive cases *vs* negative cases

^l^Fisher’s exact test, *P* < 0.001, negative cases *vs* false negative cases

^m^Fisher’s exact test, *P* = 0.491, positive cases *vs* false negative cases

^n^*χ*^2^ = 50.18, *df* = 1, *P* < 0.001, positive cases *vs* negative cases

^o^Fisher’s exact test, *P* = 0.018, negative cases *vs* false negative cases

^p^Fisher’s exact test, *P* = 0.551, positive cases *vs* false negative cases

^q^*χ*^*2*^ = 56.44, *df* = 1, *P* < 0.001, positive cases *vs* negative cases

*Abbreviation*: N, total number of cases

### Diagnostic accuracy of trichuriasis

Based on six smears, 20 out of 397 students were infected with *T. trichiura*, with 5.0% prevalence (95% CI: 2.9–7.2%) (Table 3). The number of detected cases was 16 based on a single smear, with 4.0% prevalence (95% CI: 2.1–6.0%). Thus, the false negative rate was 20.0% in a single smear compared to the ‘gold’ standard. Although an increasing trend in prevalence could be seen following an increase in the number of smears, there was no significant difference. The intensity decreased from 58.0 in a single smear to 29.8 in six smears based on available smears and from 35.0 to 29.8 based on the six smears. Thus, there was an overestimation of intensity by 94.7% in a single smear compared to the ‘gold’ standard based on available smears.

**Table 3:** Diagnostic performance of the Kato-Katz method for *T. trichiura* infection

No. of smears	No. of positive cases	No. of negative cases	Prevalence (95% CI) (%)	False negative rate (%)	GMEPG of the positive cases (1)^a^	Overestimation of GMEPG (%)^b^	GMEPG of the positive cases (2)^c^	GMEPG of the false negative cases^c^
1 stool × 1 smear	16	381	4.0 (2.1–6.0)	20.0	58.0	94.7	35.0	15.7^d^
1 stool × 2 smears	17	380	4.3 (2.3–6.3)	15.0	44.7	49.9	32.0	19.7^e^
1 stool × 3 smears	18	379	4.5 (2.5–6.6)	10.0	36.7	23.3	30.8	21.9^f^
2 stools × 1 smear	18	379	4.5 (2.5–6.6)	10.0	37.4	25.5	33.2	11.3^g^
2 stools × 2 smears	20	377	5.0 (2.9–7.2)	0.0	32.0	7.4	29.8	–
2 stools × 3 smears	20	377	5.0 (2.9–7.2)	0.0	29.8	0.0	29.8	–

^a^Only the available smears were calculated

^b^The calculation was based on the GMEPG of the positive cases (1)

^c^All six smears in ‘gold’ standard were calculated

^d^*t*_(18)_ = 1.14, *P* = 0.270, compared to the GMEPG of the positive cases (2)

^e^*t*_(18)_ = 0.60, *P* = 0.554, compared to the GMEPG of the positive cases (2)

^f^*t*_(18)_ = 0.35, *P* = 0.727, compared to the GMEPG of the positive cases (2)

^g^*t*_(18)_ = 1.15, *P* = 0.264, compared to the GMEPG of the positive cases (2)

Furthermore, we observed a significantly negative linear relationship between the prevalence and the intensity of the positive cases based on available smears (*R*^2^ = 0.862, *P* = 0.007) (Fig. 1c). However, no correlation was demonstrated between the prevalence and intensity of the false negative cases based on six smears (*R*^2^ = 0.001, *P* = 0.977) (Fig. 1d).

## Discussion

Research on human liver flukes, including *C. sinensis*, lags behind, including the evaluation of diagnostic techniques which needs to be urgently strengthened [23–25]. The present study demonstrates the significant underestimation of clonorchiasis prevalence by the Kato-Katz method when a limited number of smears are employed in a natural setting with low endemicity. The prevalence of clonorchiasis by a single smear underestimated over 40% compared to that determined by six smears from two stool samples. The diagnostic performance increased following efforts to increase the number of smears and stool samples. These findings are consistent with other helminth studies [15–19], and also with an earlier study that evaluated drug efficacy on *C. sinensis* carried out by our team [20]. Applying six Kato-Katz thick smears and two examinations by the formalin-ether concentration technique from two stool samples, 38 out of 74 individuals investigated were found to have *C. sinensis* [20]. However, only 23 were detected in a single smear, 29 in three smears and 35 in six smears. Compared with six smears, the underestimation rate was 34.3% (12/35) in a single smear, which is similar to findings (41.6%) in this study. It is not surprising that we observed a negative linear relationship between prevalence and infection intensity of detected cases, because more cases with a lower intensity could be detected following an increase in the number of smears and thus the overall intensity will decrease. Although the difference was not significant, there was an observed trend of a negative relationship between the prevalence and intensity in the false negative cases. It can be argued that the intensity of the newly detected cases through increasing the number of smears should be higher than those cases which are still false negative.

Eating raw freshwater fish correlates with *C. sinensis* infection [1]. To our knowledge, in this study, we first demonstrated the difference of eating practices between different populations by their infection status (the positive cases, negative cases and false negative cases) based on different diagnostic standards. The proportion eating raw freshwater fish was similar in two groups, namely the positive and false negative cases, which were both significantly higher than the negative cases group. This finding justifies that the increased prevalence is owing to the increased number of smears which enhances diagnostic capacity, other than factors such as contamination or low specificity.

We also demonstrated that increasing the number of smears could improve the diagnostic accuracy of *T. trichiura* infection, which is consistent with other studies [16, 18]. The difference was, however, not significant in this study, due to an extreme low prevalence. The prevalence of *C. sinensis* (19.4%) was nearly four times greater than the prevalence of *T. trichiura* (5.0%); the intensity of the former was also higher than the latter. However, underestimation in terms of prevalence in a limited number of Kato-Katz smears was still higher in *C. sinensis* than in *T. trichiura*. First, the different size and morphology of eggs are important contributors. Eggs of *C. sinensis* are smaller, measuring 27–35 × 11–20 µm, while *T. trichiura* eggs measure 50–55 × 20–25 µm [26]. Furthermore, *T. trichiura* eggs possess a pair of polar “plugs” at both ends, which are easy to distinguish [26]. Secondly, the adults of *T. trichiura* parasitize the intestines, while *C. sinensis* adults live in the biliary system [1, 27, 28]. The eggs of *C. sinensis* enter the stool with bile before they are discharged. Thus, this may impact the characteristics of the egg distribution in stools.

This study has several implications for clonorchiasis control. First, the application of multiple samples for diagnostic accuracy should be considered when individuals are reported negative with single smear sample, especially when the individual is highly suspected of being infected, e.g. when the person indulges in eating raw freshwater fish. Secondly, multiple samples are compulsory in drug evaluation, else the cure rate will be definitely overestimated. Thirdly, only a strict diagnosis can verify elimination of the endemicity of clonorchiasis. Fourthly, using a limited number of smears for surveys or surveillance activities in clonorchiasis low endemicity areas will significantly underestimate disease prevalence. Furthermore, taking into consideration the observed low intensity of the disease burden in children and females resulting from a lower frequency of eating raw freshwater fish [29], it is expected that underestimation in these groups will be high, hence, the need to pay attention to these groups. Although using a higher number of replicate samples require more resources, the effort is worthwhile because precise definitive diagnosis benefits subsequent treatment. There is, however, a challenge with the compliance and logistics when a higher number of replicate samples is collected for epidemiological surveys and surveillance. Thus, a correction factor should be introduced to estimate a real prevalence, which therefore needs more in-depth studies.

There are some limitations in this study. First, a challenge exists in distinguishing *C. sinensis* eggs from minute intestinal flukes by the Kato-Katz method [1]. Although clonorchiasis is believed to be endemic in the study area [21], we could not completely rule out the possibility of infections with other flukes in some cases. Secondly, it was found that the variation between fecal samples was greater than between smears of *Schistosoma mekongi*, but this was not demonstrated in *O. viverrini* [19]. Findings from this study reported a 16.1% prevalence in a single smear from each of two samples, while it was 12.3% in two smears from a single sample. However, the difference was not statistically significant; thus, there is need for more studies on this topic to generate more insights.

## Conclusions

The prevalence of *C. sinensis* infection is significantly underestimated, while the infection intensity is overestimated in low endemicity areas or populations because of the low accuracy of the Kato-Katz method with one fecal sample. Although a higher number of smears and samples require more resources, this deserves because it can make definite diagnosis for individuals who ate raw freshwater fish, accurately evaluate the drug efficacy and verify the elimination of endemicity. Additionally, when it is not logistically possible for multiple samples to be taken for diagnosis in surveys and surveillance in areas or populations of low endemicity, prevalence accuracy needs to be corrected.

## Data Availability

All data supporting the findings of this study are included in the article.

## Abbreviations

95% CI: 95% confidence intervals
EPG: eggs per gram of feces
GMEPG: geometric mean of eggs per gram of feces

## References

[1] Qian MB, Utzinger J, Keiser J, Zhou XN (2016). Clonorchiasis. Lancet.

[2] Qian MB, Chen YD, Liang S, Yang GJ, Zhou XN (2012). The global epidemiology of clonorchiasis and its relation with cholangiocarcinoma. Infect Dis Poverty.

[3] Chen YD, Zhou CH, Xu LQ (2012). Analysis of the results of two nationwide surveys on *Clonorchis sinensis* infection in China. Biomed Environ Sci.

[4] Jeong YI, Shin HE, Lee SE, Cheun HI, Ju JW, Kim JY (2016). Prevalence of *Clonorchis sinensis* infection among residents along 5 major rivers in the Republic of Korea. Korean J Parasitol.

[5] Doanh PN, Nawa Y (2016). *Clonorchis sinensis* and *Opisthorchis* spp. in Vietnam: current status and prospects. Trans R Soc Trop Med Hyg.

[6] Tang ZL, Huang Y, Yu XB (2016). Current status and perspectives of *Clonorchis sinensis* and clonorchiasis: epidemiology, pathogenesis, omics, prevention and control. Infect Dis Poverty.

[7] Bouvard V, Baan R, Straif K, Grosse Y, Secretan B, El Ghissassi F (2009). A review of human carcinogens—part B: biological agents. Lancet Oncol.

[8] Qian MB, Chen YD, Fang YY, Xu LQ, Zhu TJ, Tan T (2011). Disability weight of *Clonorchis sinensis* infection: captured from community study and model simulation. PLoS Negl Trop Dis.

[9] Qiao T, Ma RH, Luo XB, Luo ZL, Zheng PM (2012). Cholecystolithiasis is associated with *Clonorchis sinensis* infection. PLoS ONE.

[10] WHO. Foodborne trematode infections. http://www.who.int/foodborne_trematode_infections/en/. Accessed 2 May 2019.

[11] Hong ST, Choi MH, Kim CH, Chung BS, Ji Z (2003). The Kato-Katz method is reliable for diagnosis of *Clonorchis sinensis* infection. Diagn Microbiol Infect Dis.

[12] Choi MH, Ge T, Yuan S, Hong ST (2005). Correlation of egg counts of *Clonorchis sinensis* by three methods of fecal examination. Korean J Parasitol.

[13] Choi MH, Park SK, Li Z, Ji Z, Yu G, Feng Z (2010). Effect of control strategies on prevalence, incidence and re-infection of clonorchiasis in endemic areas of China. PLoS Negl Trop Dis.

[14] Qian MB, Yap P, Yang YC, Liang H, Jiang ZH, Li W (2013). Efficacy and safety of tribendimidine against *Clonorchis sinensis*. Clin Infect Dis.

[15] Lin DD, Liu JX, Liu YM, Hu F, Zhang YY, Xu JM (2008). Routine Kato-Katz technique underestimates the prevalence of *Schistosoma japonicum*: a case study in an endemic area of the People’s Republic of China. Parasitol Int.

[16] Nikolay B, Brooker SJ, Pullan RL (2014). Sensitivity of diagnostic tests for human soil-transmitted helminth infections: a meta-analysis in the absence of a true gold standard. Int J Parasitol.

[17] Sayasone S, Utzinger J, Akkhavong K, Odermatt P (2015). Repeated stool sampling and use of multiple techniques enhance the sensitivity of helminth diagnosis: a cross-sectional survey in southern Lao People’s Democratic Republic. Acta Trop.

[18] Liu C, Lu L, Zhang L, Bai Y, Medina A, Rozelle S (2017). More poop, more precision: improving epidemiologic surveillance of soil-transmitted helminths with multiple fecal sampling using the Kato-Katz technique. Am J Trop Med Hyg.

[19] Lovis L, Mak TK, Phongluxa K, Aye Soukhathammavong P, Vonghachack Y, Keiser J (2012). Efficacy of praziquantel against *Schistosoma mekongi* and *Opisthorchis viverrini*: a randomized, single-blinded dose-comparison trial. PLoS Negl Trop Dis.

[20] Qian MB, Yap P, Yang YC, Liang H, Jiang ZH, Li W (2013). Accuracy of the Kato-Katz method and formalin-ether concentration technique for the diagnosis of *Clonorchis sinensis*, and implication for assessing drug efficacy. Parasites Vectors.

[21] Duan JH, Tang XY, Wang QZ, Tang Y, Zhang ZS, Li ZX (2009). Epidemiological survey on clonorchiasis sinensis in an endemic area of south Hunan Province. Chin J Parasitol Parasit Dis.

[22] Katz N, Chaves A, Pellegrino J (1972). A simple device for quantitative stool thick-smear technique in *Schistosomiasis mansoni*. Rev Inst Med Trop Sao Paulo.

[23] McCarthy JS, Lustigman S, Yang GJ, Barakat RM, Garcia HH, Sripa B (2012). A research agenda for helminth diseases of humans: diagnostics for control and elimination programmes. PLoS Negl Trop Dis.

[24] Utzinger J (2012). A research and development agenda for the control and elimination of human helminthiases. PLoS Negl Trop Dis.

[25] Qian MB, Zhou XN (2019). Human liver flukes in China and ASEAN: Time to fight together. PLoS Negl Trop Dis.

[26] CDC. DPDx—Laboratory identification of parasites of public health concern. https://www.cdc.gov/dpdx/index.html. Accessed 2 May 2019.

[27] Jourdan PM, Lamberton PHL, Fenwick A, Addiss DG (2018). Soil-transmitted helminth infections. Lancet.

[28] Kim TI, Yoo WG, Kwak BK, Seok JW, Hong SJ (2011). Tracing of the Bile-chemotactic migration of juvenile *Clonorchis sinensis* in rabbits by PET-CT. PLoS Negl Trop Dis.

[29] Qian MB, Chen YD, Fang YY, Tan T, Zhu TJ, Zhou CH (2013). Epidemiological profile of *Clonorchis sinensis* infection in one community, Guangdong, People’s Republic of China. Parasites Vectors.

